# Enasidenib: An Oral IDH2 Inhibitor for the Treatment of Acute Myeloid Leukemia

**Published:** 2018-05-01

**Authors:** Rebecca A. Myers, Scott Wirth, Sherry Williams, Patrick J. Kiel

**Affiliations:** 1 Indiana University Simon Cancer Center–IU Health, Indianapolis, Indiana;; 2 University of Illinois at Chicago Cancer Center and University of Illinois at Chicago College of Pharmacy, Chicago, Illinois;; 3 Department of Pharmacy, The James Cancer Hospital and Solove Research Institute, Columbus, Ohio;; 4 Indiana University School of Medicine, Indianapolis, Indiana

## Abstract

Acute myeloid leukemia (AML) is a hematologic malignancy that affects predominantly older patients, with a median age of diagnosis around 67. Overall prognosis is poor; however, novel targeted therapies that can potentially improve outcomes in these patients have emerged in recent years. Mutations in isocitrate dehydrogenase (IDH) occur in 20% of AML diagnoses. IDH2 performs a crucial role in cellular metabolism, and when this enzyme is inhibited, the cell cannot rid itself of endogenous products and is thus marked for apoptosis. The US Food and Drug Administration (FDA) approved the first mutant IDH2 inhibitor, enasidenib, for patients with relapsed or refractory *IDH2*-mutated AML detected by an FDA-approved test.

Acute myeloid leukemia (AML) is characterized by infiltration of the bone marrow, blood, and other tissues by proliferative, clonal, abnormally differentiated, and occasionally poorly differentiated cells of the hematopoietic system. The incidence of AML has increased from 3.4 per 100,000 individuals in 2004 to 5.1 per 100,000 individuals in 2013, while death rates have unfortunately remained unchanged (American Cancer Society, 2017). Standard treatment consists of an induction phase with cytarabine plus an anthracycline, followed by a consolidation phase with high-dose cytarabine, intermediate-dose cytarabine, or allogeneic stem cell transplant depending on patient-specific factors (Döhner, Weisdorf, & Bloomfield, 2015; National Comprehensive Cancer Network, 2017).

Acute myeloid leukemia is a complex hematologic malignancy that is driven by multiple genetic mutations, including isocitrate dehydrogenase 2 (*IDH2*; Medinger & Passweg, 2017). *IDH2* is an enzyme of the citric acid cycle, and when mutated alters DNA methylation leading to impaired cellular differentiation (Ragon & DiNardo, 2017). Although the cytogenetic heterogeneity of this disease has been recognized for over 30 years, the extensive molecular heterogeneity of AML has come into realization within the past decade (Medinger & Passweg, 2017). The prognostic importance of this heterogeneity has been well accepted, but it is only recently that this information coupled with advancing technology could be translated into new treatment options (Nassereddine, Lap, Haroun, & Tabbara, 2017). The *IDH2* mutations prevent blasts in the bone marrow from differentiating into mature functioning blood cells, and it is estimated that 12% of adults with AML may have such mutations (Stein et al., 2017). Although other cytogenetic and molecular aberrations have been associated with inferior outcomes, the prognostic implication of the *IDH2* mutation remains unclear.

Enasidenib (Idhifa), which targets *IDH2*, was granted approval by the US Food and Drug Administration (FDA) in 2017 for the treatment of adult patients with relapsed or refractory AML with an *IDH2* mutation as detected by an FDA-approved test (Celgene Corporation, 2017).

## MECHANISM OF ACTION

**Figure 1:**
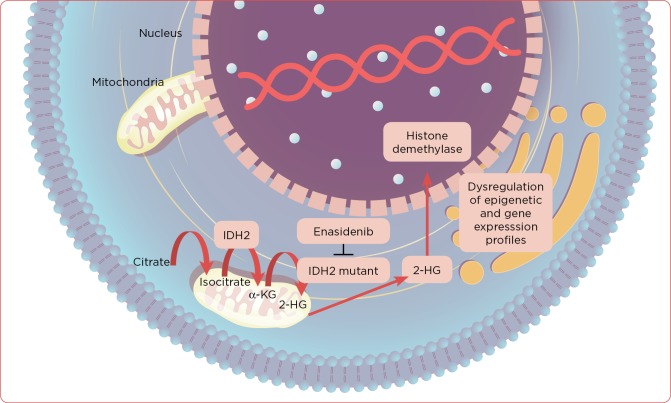
Mutated IDH2 proteins synthesize 2-hydroxyglutarate, an oncometabolite, which results in DNA and histone hypermethylation and blocks myeloid differentiation. IDH2 = isocitrate dehydrogenase 2; α-KG = alpha-ketoglutarate; 2-HG = 2-hydroxyglutarate.

Mutated IDH2 proteins synthesize 2-hydroxyglutarate (2-HG), an oncometabolite, which results in DNA and histone hypermethylation and blocks myeloid differentiation (Figure 1; Nassereddine et al., 2017). Enasidenib is a novel, oral, small-molecule inhibitor of the IDH2 enzyme. Specifically, it targets mutant IDH2 variants R140Q, R172S, and R172K, leading to decreased serum 2-HG levels and therefore an increased rate of myeloid differentiation and reduced blast counts (Celgene Corporation, 2017).

## CLINICAL TRIALS

Stein and colleagues (2017) evaluated enasidenib in a phase I/II clinical trial to evaluate the maximum tolerated dose, pharmacokinetics and pharmacodynamics, safety profile, and clinical efficacy. The trial included 239 adults with *IDH2*-mutated relapsed/refractory AML or myelodysplastic syndromes with refractory anemia with excess blasts (RAEB), with AML patients representing the largest subgroup in the study (74% of all patients; Stein et al., 2017). During the dose escalation phase, patients received enasidenib either daily (dose range, 50–650 mg) or twice daily (dose range, 30–150 mg) in continuous 28-day cycles. A dose of 100 mg daily was chosen for the dose expansion phase based on achieved steady-state concentrations, plasma 2-HG reduction, and clinical efficacy.

The overall response rate (ORR) was 40.3%, with 34 patients (19.3%) achieving a complete remission. Median time to first response was 1.9 months (range, 0.5–9.4 months), with 87.3% patients obtaining a response by cycle 5. Clinical activity was observed in patients with *IDH2* mutations R140 and R172 (ORR 35.4% and 53.3%, respectively) and 2-HG suppression from baseline to cycle 2; day 1 was not correlated with clinical response. Median overall survival (OS) was 9.3 months (95% confidence interval [CI] = 8.2–10.9 months), and estimated 1-year survival was 39%. For patients achieving a complete or partial remission, median OS was 19.7 months and 14.4 months, respectively. Median event-free survival duration was 6.4 months (95% CI = 5.4–7.5 months), and 30-day mortality was 5.1% (Stein et al., 2017). Of the 157 patients who were red blood cell and/or platelet transfusion dependent prior to study entry, 34% achieved transfusion independence.

## ADVERSE EFFECTS

Treatment-related adverse events were observed in 82% (195 of 239) of patients in the phase I/II study (Table 1). Treatment-related grade 3 or 4 adverse events occurred in 41% of patients (Table 2). The most common serious adverse events included IDH-inhibitor–associated differentiation syndrome (IDH-DS; 8%) and leukocytosis (4%). Dose modification, interruption, or discontinuation due to treatment-related adverse events was required in 7%, 22%, and 5% of patients, respectively (Stein et al., 2017).

**Table 1:**
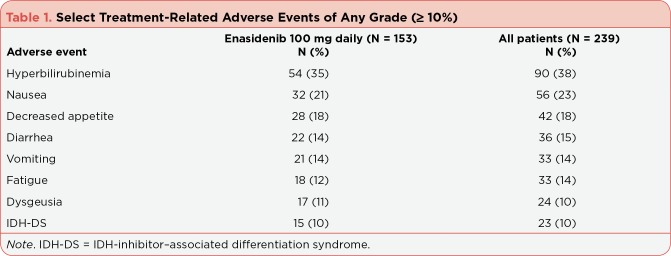
Select Treatment-Related Adverse Events of Any Grade (≥ 10%)

**Table 2:**
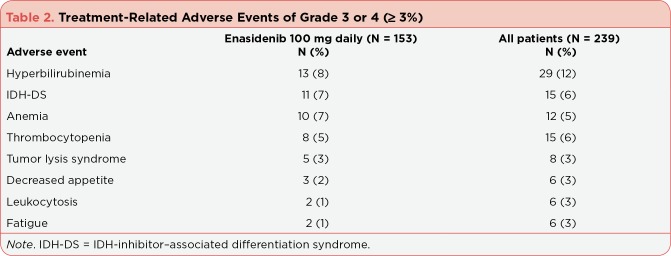
Treatment-Related Adverse Events of Grade 3 or 4 (≥ 3%)

Enasidenib’s FDA label includes a black box warning for IDH-DS, which, if left untreated, may be life-threatening or fatal. Signs and symptoms of IDH-DS include fever, dyspnea, pulmonary infiltrates, peripheral edema accompanied by rapid weight gain, and acute renal failure. In the phase I/II trial, IDH-DS of any grade occurred in 23 patients (15 patients with grade 3 or 4) and the median onset was 48 days (range, 10–340 days). Management consisted of systemic corticosteroids (19 of 23 patients), required dose interruption in 10 patients, and no patients required permanent drug discontinuation. Enasidenib may also lead to rapid myeloid proliferation that presents as noninfectious leukocytosis. Treatment-related leukocytosis occurred in 6% of patients (6 patients with grade 3 or 4), lead to study discontinuation in 1 patient, and dose interruption in 6 patients (Celgene Corporation, 2017; Stein et al., 2017).

## DOSING AND ADMINISTRATION

Enasidenib is approved for adult patients with relapsed or refractory AML with an *IDH2* mutation detected by an FDA-approved test. The recommended starting dose of enasidenib is 100 mg orally once daily with or without food until disease progression or unacceptable toxicity. For patients without disease progression or unacceptable toxicity, it is recommended to treat for a minimum of 6 months to allow time for a clinical response. Blood counts and blood chemistries should be assessed prior to initiation and every 2 weeks for the first 3 months during treatment.

Patients should not chew or split the tablets, but swallow tablets whole. If a patient misses or vomits after a dose of enasidenib, he/she should take it as soon as possible on the same day and return to the normal schedule the following day.

## IMPLICATIONS FOR THE ADVANCED PRACTITIONER

Enasidenib is a novel treatment option for patients with relapsed or refractory AML harboring an *IDH2* mutation. Although not directly compared to other therapies in the relapsed or refractory setting, enasidenib may provide a response, create or maintain transfusion independence, allow transition to hematopoietic stem cell transplant, and/or improve survival in heavily pretreated patients. In order to receive these potential benefits, it is important that the advanced practitioner assist in detecting *IDH2* mutations throughout the course of the disease in patients with AML. Enasidenib’s potential efficacy is specific for those with *IDH2* mutations; a number of testing strategies are available, including an FDA-approved polymerase chain reaction–based companion diagnostic test. It is also important that the advanced practitioner understand the potential adverse effects that could result from the use of enasidenib.

Although enasidenib was studied at much higher doses in the phase I approval study (Stein et al., 2017), the recommended dose of enasidenib is 100 mg orally once daily until disease progression and/or toxicity (Celgene Corporation, 2017). There are no defined dose adjustments for renal dysfunction, but the dose should be reduced for an elevated bilirubin of greater than 3 times the upper limit of normal that is sustained for 2 weeks or longer without elevated transaminases or other hepatic disorders.

The most common serious side effects to enasidenib include nausea, vomiting, diarrhea, tumor lysis syndrome, leukocytosis (often noninfectious), and decreased appetite (Celgene Corporation, 2017; Stein et al., 2017). Unlike cytotoxic chemotherapy, myelosuppression is uncommon. Other clinically significant side effects include electrolyte disturbances (hypocalcemia, hypokalemia, hypophosphatemia), hyperbilirubinemia, and differentiation syndrome.

Although differentiation syndrome occurred at a fairly low incidence (14%), it is important that the advanced practitioner understand the monitoring and management of this complication, as it is life-threatening. Common symptoms may include shortness of breath, lymphadenopathy, fever, rapid weight gain, and multi-organ dysfunction (including hepatic and renal dysfunction). Differentiation syndrome may be delayed anywhere from 10 days to 5 months following initiation of treatment. It is imperative that advanced practitioners educate patients on the potential signs and symptoms and instruct patients to seek medical care immediately, especially if they are taking the medication at home or away from the medical team. Systemic corticosteroids and hemodynamic monitoring should be initiated immediately upon diagnosis. Enasidenib does not always need to be discontinued for differentiation syndrome, but it may be for severe complications (Table 3).

**Table 3:**
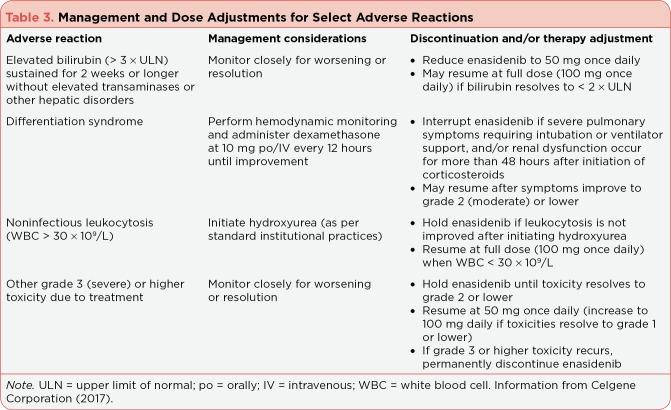
Management and Dose Adjustments for Select Adverse Reactions

Dose adjustments for hyperbilirubinemia have been described previously and it should be considered that this adverse effect is often temporary and a result of off-target inhibition of UGT1A1 by enasidenib. Each patient should be assessed clinically, but often only the indirect bilirubin is elevated and of minimal clinical consequence. The incidence of noninfectious leukocytosis in patients on enasidenib may complicate a practitioner’s ability to assess response and/or progression of disease; it is important to assess patients on a case-by-case basis. If it is determined the leukocytosis is due to the enasidenib, hydroxyurea may be initiated per standard institutional practices. Enasidenib is an inhibitor of multiple cytochrome P (CYP) enzymes (CYP1A2, CYP2B6, CYP2C8, CYP2C9, CYP2C19, CYP2D6, CYP3A4). It also inhibits UGT1A1 (primary off-target effect responsible for hyperbilirubinemia) and P-glycoprotein. It also induces CYP2B6 and CYP3A4. The clinical implications of these interactions are unknown due to the lack of formal drug-drug interaction studies. However, the current prescribing information suggests that alterations (increase or decrease) of the concentration of combined oral contraceptives may occur (Celgene Corporation, 2017).

Enasidenib is currently being evaluated in combination regimens with agents such as azacitidine or induction chemotherapy in patients with newly diagnosed *IDH2*-mutated AML (Agios Pharmaceuticals, Inc., 2017). These studies are evaluating safety and efficacy and are currently still in the recruitment stages. Further detection studies are underway as well, looking to detect *IDH1* and *IDH2* mutations in other types of cancer such as glioma (Agios Pharmaceuticals, Inc., 2017).

## CONCLUSION

Historically, patients with AML have had limited treatment options that mostly included intravenous cytotoxic chemotherapy. The approval of enasidenib provides a novel targeted oral treatment option for those harboring an *IDH2* mutation with fewer myelosuppressive side effects when compared to chemotherapy. Given this novel class of medication, the advanced practitioner will need to provide medication education, side-effect management, appropriate monitoring, and follow-up assessment to patients with *IDH2*–mutated AML.
